# *SMAXI*: a machine-learning-powered open-source software for multidimensional full-field X-ray image analysis

**DOI:** 10.1107/S1600577526007745

**Published:** 2026-08-26

**Authors:** Dukyong Kim, Tao Sun

**Affiliations:** a Department of Mechanical Engineering Northwestern University Evanston IL USA; Australian Synchrotron, Australia

**Keywords:** full-field X-ray imaging, open-source software, machine learning, large language model

## Abstract

Full-field X-ray imaging techniques using laboratory-based and synchrotron sources are powerful and versatile tools used by academia and various industries to non-destructively characterize the internal structures of samples. In particular, synchrotron high-speed radiography and computed tomography (CT) have helped researchers address many critical problems by providing multiscale and multidimensional structural information. Owing to increased source brightness and improved spatial and temporal resolutions of modern detection systems, the generation rate of X-ray imaging data has accelerated dramatically in recent years. Consequently, manually analyzing the massive volume of X-ray image data has become a persistent bottleneck that hinders fast scientific discoveries and data-driven insights. To address this challenge, we developed *SMAXI* (*Software for Machine-Learning-assisted Analysis of X-ray Images*), an open-source software designed for analyzing multidimensional full-field X-ray image data. *SMAXI* distinguishes itself from other conventional closed-source software by being fully customizable and open-source. It is GPU-accelerated and uses state-of-the art machine learning (ML) models to analyze time-resolved multidimensional X-ray imaging data. *SMAXI*’s capabilities for image processing and analysis are demonstrated here using 2D static X-ray images, dynamic *in situ*X-ray videos, and complex volumetric CT data. By integrating advanced ML algorithms into a single customizable workflow, *SMAXI* can pre-process X-ray images using computer vision algorithms and automate object segmentation and tracking tasks, while utilizing a large language model-based chatbot for interactive geometry feature analysis. Ultimately, *SMAXI* will empower X-ray community members to overcome big-data limitations, accelerating the pace of scientific discovery that incorporates full-field X-ray imaging techniques.

## Abstract 2

*SMAXI* is a fully open-source, GPU-accelerated software platform that integrates state-of-the-art machine learning and large language model techniques into a unified workflow for X-ray image analysis. *SMAXI* simplifies high-throughput data processing workflows and overcomes manual image analysis bottlenecks.

## 1. Introduction

Full-field X-ray imaging is a group of non-destructive imaging techniques wherein an X-ray beam penetrates a specimen, projecting its 2D attenuation map onto an area detector where the resulting spatial intensity distribution is captured as a pixel-wise digital image (Shen *et al.*, 2007 ▸). Full-field X-ray imaging is utilized widely across various fields in both academia and industry due to its ability to reveal internal structures and their dynamic evolution under external stimuli (Ou *et al.*, 2021 ▸; Lim *et al.*, 2016 ▸; Cunningham *et al.*, 2019 ▸). For instance, high-speed X-ray radiography has been used to visualize defect generation in additive manufacturing (AM) with high temporal resolution (Cunningham *et al.*, 2019 ▸; Zhao *et al.*, 2020 ▸). Nano-computed tomography (CT) is employed by the semiconductor industry to characterize integrated circuit samples for quality control in the chip fabrication process (Aidukas *et al.*, 2024 ▸; Holler *et al.*, 2017 ▸). For fundamental research, full-field X-ray imaging techniques in different contrast-forming modes are often utilized for the 3D characterization of novel energy materials (Cao *et al.*, 2020 ▸), nanostructures (Rand *et al.*, 2011 ▸), and the mapping of neural networks in the human brain (Chin *et al.*, 2020 ▸), to name a few.

The broad application of full-field X-ray imaging techniques is enabled by the availability of a wide range of small- and large-scale instruments. The commercial laboratory-scale X-ray imaging systems are well suited for *ex situ* CT and certain *in situ* experiments that do not involve rapid structural dynamics, due to their low source brightness. In this work, the term *in situ* specifically refers to time-resolved, operando imaging datasets, and *ex situ* refers to static, non-time-resolved datasets. In contrast, the synchrotron-based radiography and CT beamlines, with much higher beam flux, are ideal for high-throughput *ex situ* experiments and *in situ* characterization that demands high spatial and temporal resolutions (Singh *et al.*, 2024 ▸).

The deployment of commercial X-ray CT systems has grown steadily in recent years, driven by increasing demand for high-resolution, non-destructive testing across industries such as aerospace, automotive, and semiconductor manufacturing. Meanwhile, source brightness at synchrotron facilities continues to increase, alongside the adoption of high-resolution, high-efficiency detectors (Nikitin, 2023 ▸). Collectively, the data generation rate has been increasing substantially in recent years. These advances have substantially accelerated data generation rates. For example, a single three-day beamtime at a CT beamline can generate more than one million X-ray images. Consequently, post-experiment image processing and analysis have become significant bottlenecks (Gürsoy *et al.*, 2014 ▸). For many researchers, manually labeling structural features on the X-ray images is either too time-consuming for large datasets or too technically challenging, particularly when the images exhibit relatively low contrast and instrument-induced artifacts (Adams *et al.*, 2021 ▸; Wang *et al.*, 2018 ▸).

To overcome these bottlenecks, researchers are increasingly turning to image analysis software incorporating machine learning (ML) models to segment morphological details by precisely segmenting boundaries of the object of interest in X-ray datasets. Currently, image analysis methods are often provided to the X-ray imaging community through commercial software. Most of these platforms were developed for X-ray CT applications, driven by the need for developing mathematical algorithms to reconstruct complex 3D sliced images. Dragonfly (Comet, Canada) provides robust tools for image processing, segmentation and visualization, and has recently integrated ML models and graphics processing unit (GPU) acceleration for nano-CT analysis (Košek *et al.*, 2024 ▸; Yang *et al.*, 2023 ▸). Similarly, Avizo (Thermo Fisher Scientific, USA) provides material-specific solutions, featuring artificial intelligence (AI) enhanced segmentation (Sun & Zhang, 2025 ▸) for CT data (Li *et al.*, 2021 ▸). Hardware companies have also developed software, such as *INSPECT X-Ray* (ZEISS, Germany) and *Inspect-X* (Nikon, Japan), which specialize in enhanced visual non-destructive inspection of CT data.

However, most commercial software remains biased toward static CT image analysis and is often inefficient in handling the highly dynamic, time-resolved datasets characteristic of modern full-field imaging experiments. Furthermore, the closed-source architecture of these commercial tools restricts customization, and accessing their advanced ML-accelerated features typically requires subscription fees. Ultimately, these limitations highlight a critical gap between current research demands and available software tools, driving the need for an open-source, highly adaptable image analysis platform that can process multidimensional X-ray data obtained using lab-based systems and synchrotron beamlines.

To overcome the limitations of existing software and further accelerate the analysis of full-field X-ray imaging data, we introduce *SMAXI* (*Software for Machine-Learning-assisted Analysis of X-ray Images*). *SMAXI* is an open-source platform that integrates advanced ML models into a streamlined pipeline designed to pre-process, segment, track, and quantify experimental features within a unified framework. Its primary application is the rapid and automated analysis of dynamic morphological features in X-ray image datasets collected from laboratory-based systems or synchrotron facilities. Advanced ML models developed by computer vision and other research communities are leveraged to achieve high processing efficiency and fidelity. In addition, *SMAXI* integrates a large language model (LLM) interface that allows users to interactively extract quantitative data using simple query prompts such as ‘Calculate the average and standard deviation of the object’s height’. A detailed discussion of *SMAXI*’s architecture and its core functions is given in Section 2.

## 2. *SMAXI*: ML-accelerated X-ray image analysis program

**Figure 1:**
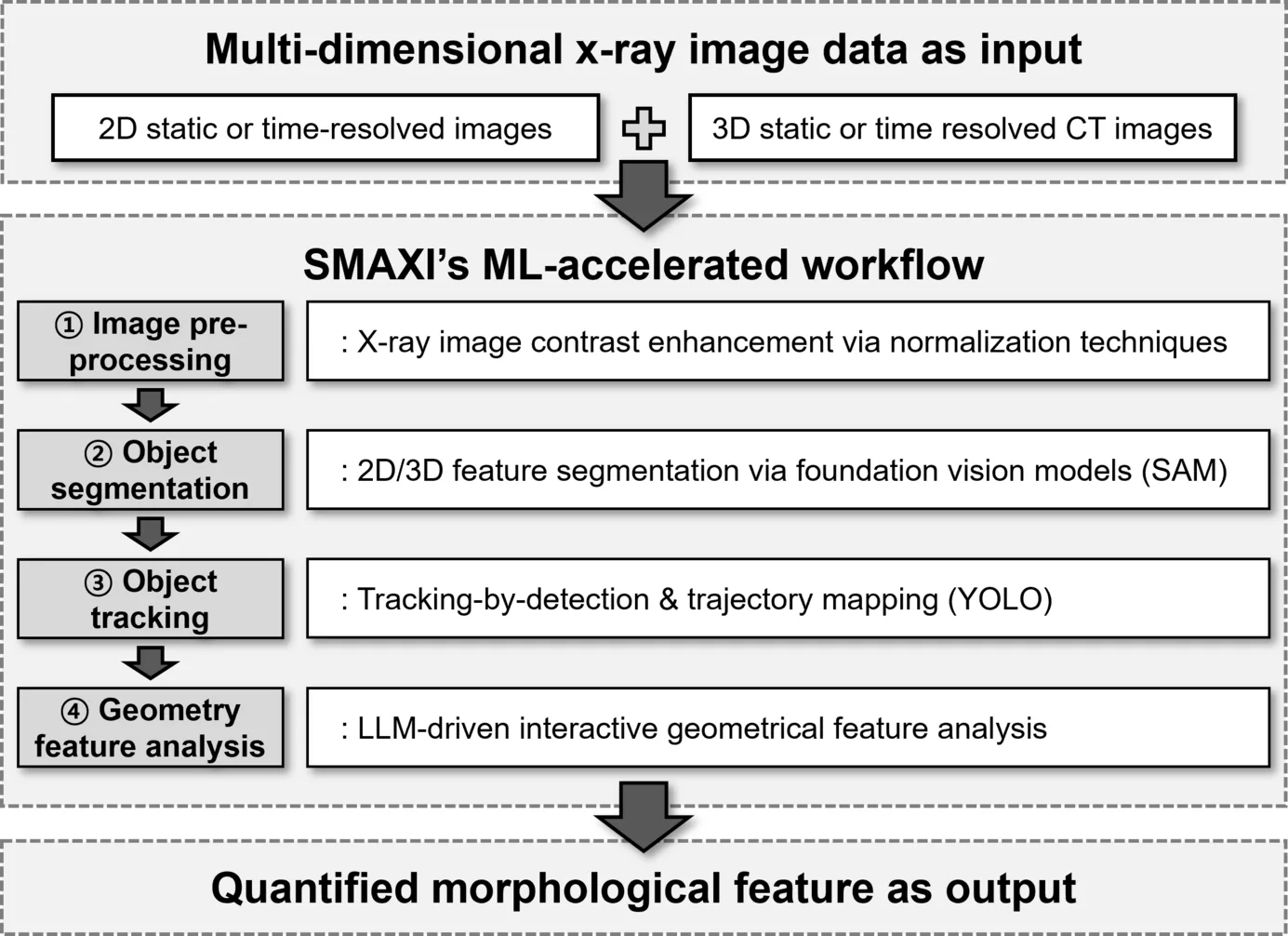
Overview of *SMAXI*’s architecture. Multidimensional raw X-ray images, ranging from static 2D X-ray images to 3D time-resolved *in situ* CT data can be utilized as input data. These data are then processed through a sequential workflow: (1) image pre-processor to enhance the image contrast through pixel normalization, (2) SAM-based object segmentation feature for annotating 2D and 3D objects, (3) YOLO-driven object detection and trajectory tracking, and (4) LLM-assisted geometrical feature analysis. Finally, the morphological features of tracked objects, such as area, perimeter, width, depth, and aspect ratio, are quantified as output.

The overall architectural design and sequential workflow of *SMAXI* are illustrated in Fig. 1 ▸. Built upon a *Tkinter* Python library GUI, *SMAXI* utilizes ML models that can run on GPU hardware to execute an accelerated multistage pipeline for analyzing X-ray images. The raw X-ray images can be high-speed radiography data, *in situ* and *ex situ* CT data. These images are processed through a series-driven workflow, structured using four modules: (1) image pre-processing feature for image contrast enhancement via background image division-based normalization, (2) an interactive object segmenter based on a foundation vision model, Segment Anything Model (SAM) (Meta, USA) (Kirillov *et al.*, 2023 ▸), (3) a temporal tracking framework utilizing You Only Look Once (YOLO) (Ultralytics, USA) tracking-by-detection algorithms to map object trajectories over time (Sohan *et al.*, 2024 ▸), and (4) a LLaMA (Meta, USA) based LLM interface for interactive and advanced natural language analysis of the extracted coordinates (Touvron *et al.*, 2023 ▸).

In standard X-ray analysis, *SMAXI* functions primarily as a post-reconstruction tool. Advanced synchrotron-based X-ray imaging methods, such as phase-contrast imaging, holography, or CT, still require computational reconstructions of raw data, which often depend on human tuning. *SMAXI* does not attempt to replace these initial physics-driven steps. Instead *SMAXI* assumes the raw detector data has already been converted into interpretable 2D radiographs. Therefore, *SMAXI*’s core purpose is to take over immediately after reconstruction to eliminate the tedious manual tasks that typically follow, such as frame-by-frame hand-tracing, which is done by polygon drawing over the object of interest. Looking ahead, *SMAXI*’s modular architecture (explained in more detail in Section 2.2.2) is expected to make it easy for the community to build custom plugins for these earlier reconstruction algorithms, which could eventually bring the entire analysis under one pipeline.

To automate these workflows, *SMAXI* integrates advanced ML models, building upon previous studies that have successfully demonstrated ML-based segmentation of X-ray image datasets. For general image segmentation purposes, convolutional neural network (CNN)-based models, such as U-Net (Ronneberger *et al.*, 2015 ▸), XNet (Bullock *et al.*, 2019 ▸), and SegNet (Badrinarayanan *et al.*, 2017 ▸) have been widely utilized. For example, in medical applications, these models have successfully been employed to segment bone structures (Cernazanu-Glavan & Holban, 2013 ▸) or bone fracture (Ghoti *et al.*, 2021 ▸). Specific to the AM domain, AM–SegNet, a custom CNN-based deep learning segmentation model, was developed for segmenting melt pool features, demonstrating superior performance over U-Net (Li *et al.*, 2024 ▸), while unsupervised Gaussian Mixture Models have been utilized for real-time bubble tracking to reveal pore dynamics and melt flow patterns (Liu *et al.*, 2025 ▸). Although these specialized approaches have demonstrated success, this research focuses on utilizing foundational models such as SAM and YOLO. The key advantage of these foundational models over U-Net and XNet is their ability to deliver high-quality, prompt-driven, zero-shot segmentation that generalizes across completely new datasets and imaging conditions without requiring any retraining, thereby significantly accelerating downstream analysis tasks (Zhang *et al.*, 2023 ▸). Recently, for example, joint SAM 1 and YOLO workflows have been implemented for automated lung segmentation in chest X-rays (Khalili *et al.*, 2024 ▸; Pandey *et al.*, 2023 ▸), and SAM 2 has been rapidly adapted across various medical imaging modalities (He *et al.*, 2026 ▸; Dong *et al.*, 2026 ▸; Ma *et al.*, 2025 ▸; Chukwujindu *et al.*, 2025 ▸; Zhang *et al.*, 2024 ▸). Therefore, by utilizing this integrated ML pipeline, *SMAXI* minimizes manual intervention, transforming raw pixel data into quantified spatiotemporal morphological metrics such as an object’s cross-sectional area, width and aspect ratio. The specific methodologies of these novel ML model-based features that were integrated into *SMAXI* are detailed in the subsequent sections.

### 2.1. Major functions of *SMAXI*

#### 2.1.1. Image contrast enhancement through gray-scale normalization

**Figure 2:**
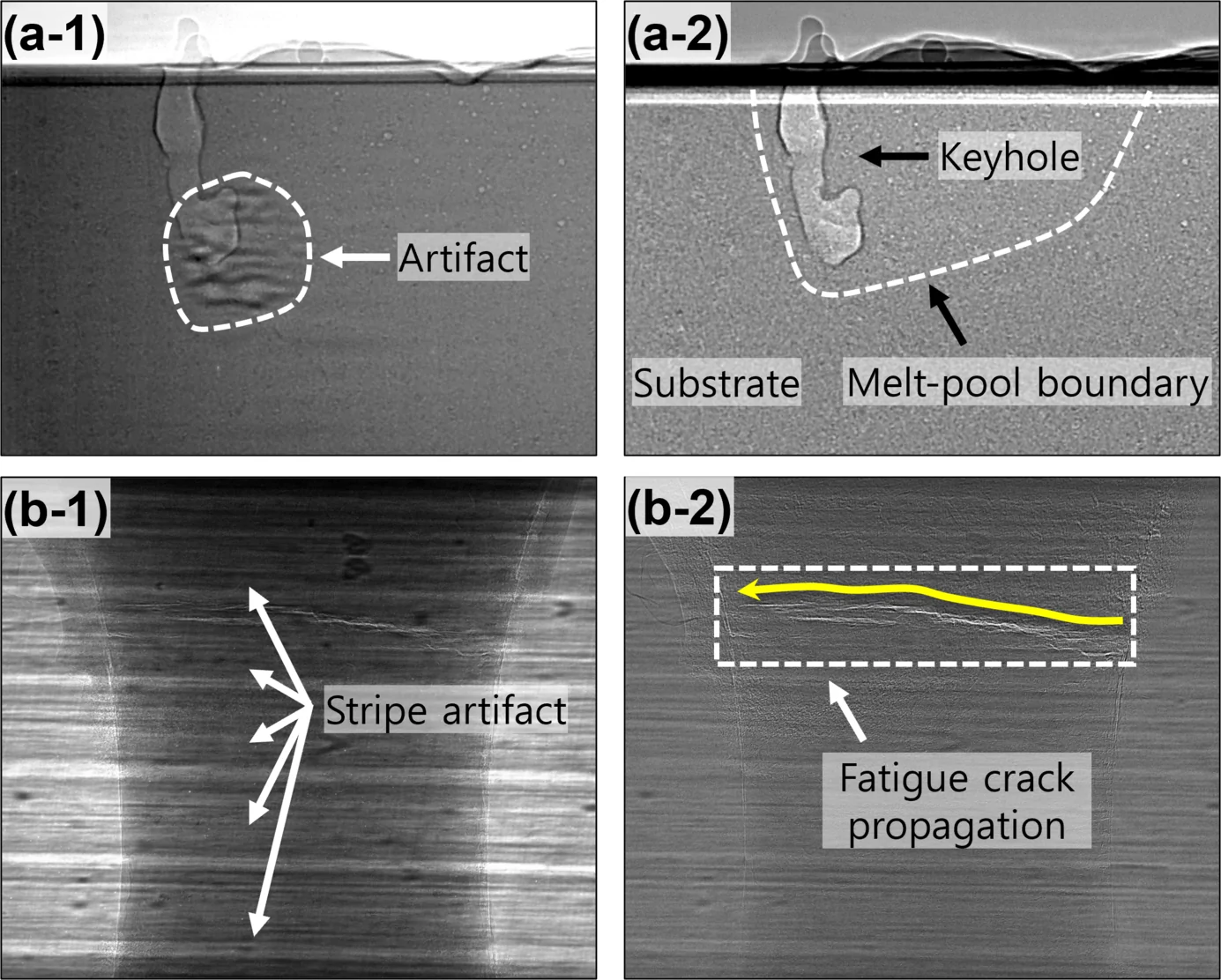
Efficacy of *SMAXI*’s gray-scale intensity normalization module. (*a*-1) A raw high-speed X-ray image of an L-PBF process of Ti-6Al-4V plate sample, exhibiting low contrast and background artifacts. (*a*-2) The normalized frame demonstrating sharpened image boundaries that clearly isolate the keyhole-shaped vapor depression and the surrounding melt pool. (*b*-1) A raw projection image of *in situ* CT experiment on fatigue corrosion of Al7075, with horizontal stripe artifacts present (Stannard *et al.*, 2017 ▸). (*b*-2) The pre-processed output, successfully flattening the illumination gradient to reveal the underlying fatigue crack propagation.

Raw X-ray image datasets frequently suffer from various radiometric artifacts, such as varying illumination gradients across pixel frames or intrinsic X-ray detector noise (Takeuchi *et al.*, 2023 ▸). To isolate dynamic features from artifacts and enhance image contrast, *SMAXI* first implements a background image division algorithm. Let *I*_raw_(*x*, *y*, *t*) denote the raw pixel intensity at spatial coordinates (*x*, *y*) at time step *t*, and let *I*_bg_(*x*, *y*) represent a static baseline background frame captured prior to the dynamic event, for example the very initial frame before a process begins, or a user-defined frame selected before the event of interest. The flat-field corrected image, *I*_div_, is calculated as follows,
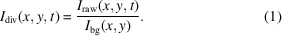
This process flattens the background, leaving only transient structural variations fully isolated, such as the evolving vapor depression cavity (a.k.a. keyhole) in the melt pool during the laser powder bed fusion (L-PBF) process shown in Fig. 2 ▸. Following background division, *SMAXI* rescales the resulting pixel intensities using a percentile-based continuous grayscale normalization method. This method is advantageous over utilizing absolute minimum and maximum intensity values, because such a binary normalization method may miss subtle pixel value changes that represent a subtle physical event, such as a cavitation event or microcrack propagation. Therefore, by utilizing a gray-scale normalization method, *SMAXI* extracts percentiles from the raw intensity distribution to define a stable contrast window, thereby applying an enhanced image contrast to a raw image dataset. It is important to note that while this normalization is required to format the images for the SAM and YOLO backbones, which expect 8-bit inputs, it inherently compresses raw pixel intensities and alters true physical values, such as X-ray attenuation. Consequently, *SMAXI* uses this normalized data exclusively to generate geometric boundaries and tracking coordinates. To compute intensity-based quantitative metrics, such as density histograms, users must apply the binary masks and coordinates exported by *SMAXI* back onto the original, un-normalized raw datasets to prevent information loss. The normalized floating-point intensity, *I*_norm_, is calculated as follows,
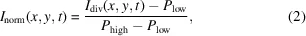
where *P*_low_ and *P*_high_ represent the lower and upper intensity percentiles, respectively, determined dynamically from the pixel intensity distribution of the individual image frame at time *t* to adaptively maximize local contrast variations. To map these normalized values into a standard digital format compatible with ML algorithms, using equation (3), an 8-bit pixel intensity for a given spatiotemporal coordinate [*I*_8bit_(*x*, *y*, *t*)] is generated,
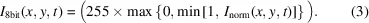
The efficacy of the continuous gray-scale normalization process is demonstrated across X-ray images acquired from different engineering applications in Fig. 2 ▸. In high-speed imaging of L-PBF process using Ti-6Al-4V plate samples, the raw frame [Fig. 2 ▸(*a*-1)] displays a poorly resolved keyhole vapor depression and melt pool boundaries caused by artifacts, Such artifacts can be introduced by the source, X-ray optics, and the detector, which are common for full-fieldX-ray imaging. By utilizing *SMAXI*’s continuous gray-scale normalization method, the radiometric contrast is dynamically equalized, sharply resolving the internal keyhole morphology and revealing the precise outline of the melt pool boundary indicated by the white dotted line [Fig. 2 ▸(*a*-2)]. Furthermore, *SMAXI* demonstrates robust performance in revealing fatigue crack of raw fatigue test of corrosion pits in Al7075 alloy [Fig. 2 ▸(*b*-1)] (Stannard *et al.*, 2017 ▸). The normalization algorithm successfully mitigates background intensity gradients and filters out the horizontal banding [Fig. 2 ▸(*b*-2)], cleanly isolating the structural path of fatigue crack propagation represented by the yellow arrow. This pre-processing module can effectively eliminate artifacts prevalent in X-ray images, thus delivering optimized data for subsequent ML-powered segmentation or feature tracking in *SMAXI*.

#### 2.1.2. Interactive segmentation via foundation vision models (SAM)

Following image pre-processing, the second step in the *SMAXI* workflow involves segmenting the boundary of the objects of interest. Building upon the foundational model advantages outlined in Section 2, *SMAXI* supports several foundation model backbones across both the SAM 1 (Kirillov *et al.*, 2023 ▸) and SAM 2 (Dong *et al.*, 2026 ▸). To maintain focus on the practical applications of *SMAXI*, the highly technical ML architecture specifications, model parameters, and algorithmic details for these foundational modules have been detailed separately in Appendix *A*[App appa].

The configurations and primary use cases of SAM 1 and SAM 2 for object segmentation are summarized in Table 1 ▸. The structural differences between these models directly impact processing speed and boundary accuracy. SAM 1 baselines rely on standard global Vision Transformer (ViT), where computational overhead scales quadratically with the number of image patches. In contrast, the SAM 2 framework utilizes a hierarchical ViT backbone (Hiera) pre-trained via masked autoencoders. By calculating spatial attention within localized windows and passing multiscale features through successive processing stages, SAM 2 substantially reduces inference latency, the total time needed for ML model to process input and return a response, thereby maintaining a reliable feature segmentation workflow.

**Figure 3:**
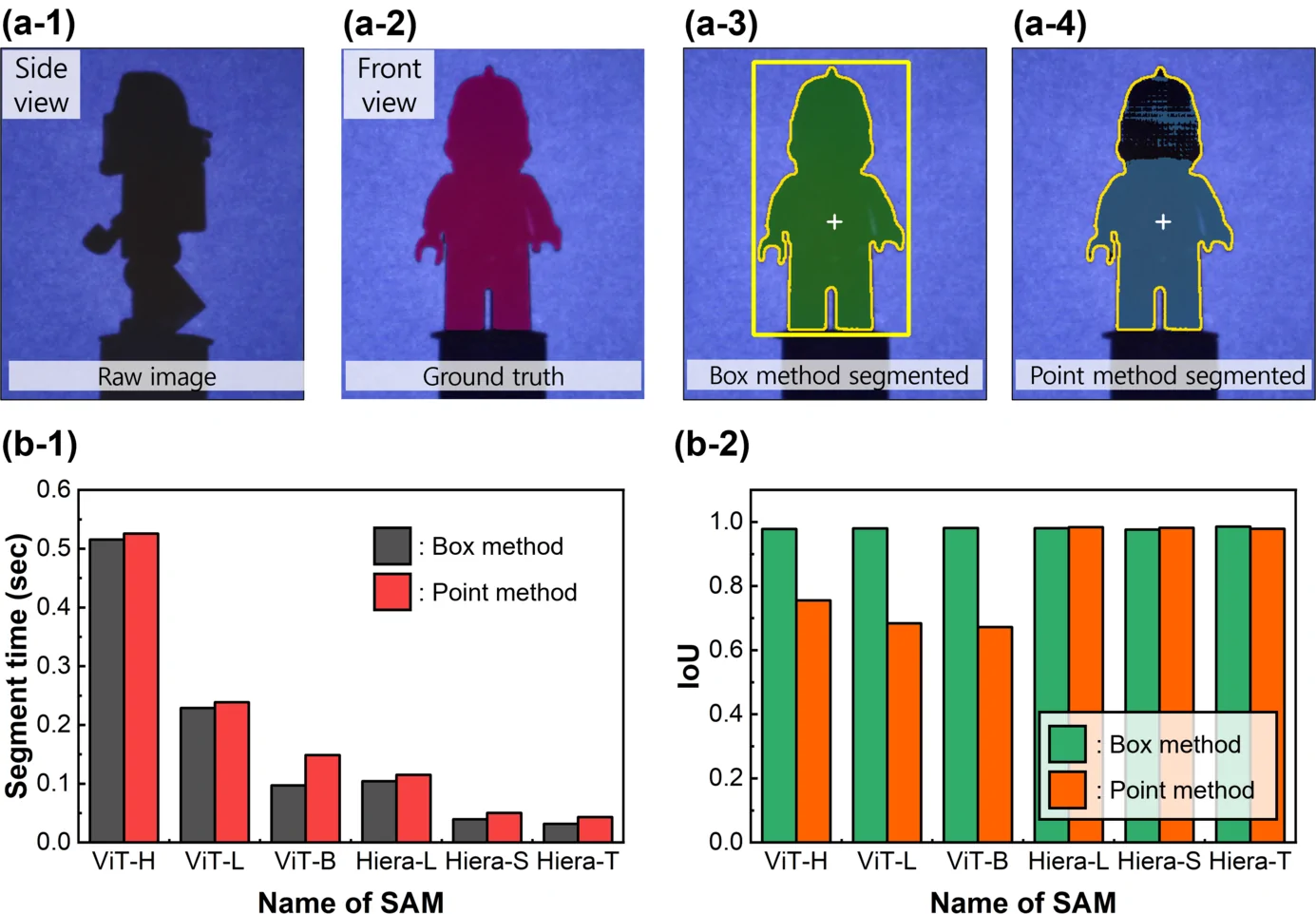
Quantitative evaluation of SAM architectures and prompting strategies within *SMAXI* using a slice of CT data of a LEGO figure (De Carlo *et al.*, 2018 ▸) (*a*-1) Raw image of the LEGO figure (side view) and (*a*-2, *a*-3, *a*-4) visual comparison of a reference ground truth mask against segmentations generated via bounding box and single-point prompts using SAM 1 (ViT-H) for the LEGO figure (front view). (*b*-1) Computation time required for segmentation across various SAM 1 (ViT-H, ViT-L, ViT-B) and SAM 2.1 (Hiera-L, Hiera-S, Hiera-T) backbones, highlighting the superior inference efficiency of the Hiera architectures. (*b*-2) Corresponding IoU scores, demonstrating that bounding box prompts consistently yield high fidelity masks (IoU > 0.95) across all architectures. While the box method significantly outperforms single-point prompting in SAM 1 backbones, the advanced Hiera architectures in SAM 2 demonstrate robust convergence, achieving near-perfect IoU scores for both prompting strategies.

The operational trade-offs governing these backbone scales and prompting strategies are quantified in Fig. 3 ▸ using CT dataset of a LEGO^®^ figure (De Carlo *et al.*, 2018 ▸), where Fig. 3 ▸(*a*-1) represents the raw image at the side view and Fig. 3 ▸(*a*-2) indicates the ground truth segmented area from the front view. As shown in the qualitative comparison using SAM 1 (ViT-H) in Figs. 3 ▸(*a*-3) and 3 ▸(*a*-4), the bounding box [Fig. 3 ▸(*a*-3)] can successfully isolate the target profile from the raw CT image. However, the single-point method [Fig. 3 ▸(*a*-4)], where the point was set to the center of the image, reveals minor boundary noise along the base, whereas the bounding box prompt matches the clean edge profile of the manual ground truth. These results are quantified and summarized in the benchmarking analysis [Fig. 3 ▸(*b*-1)]. The result reveals that the Hiera-L, Hiera-S and Hiera-T models demonstrate a significant reduction in execution latency, completing a full segmentation loop under 0.15 s, whereas the legacy ViT-H model demands over 0.5 seconds per frame. Fig. 3 ▸(*b*-2) highlights the geometric convergence behavior driven by the prompt mathematical formulation. While the bounding box prompt method drastically outperforms single-point prompting in the SAM 1 architectures, both methods achieve optimal performance with the advanced Hiera backbones of SAM 2, consistently maximizing the structural Intersection over Union (IoU > 0.95) across all architecture options. Here, IoU is calculated via the element-wise intersection and union of the predicted binary mask matrix *M*_pred_ and the reference ground-truth mask matrix *M*_gt_,
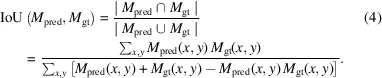
By framing the target with a bounding box, the model enforces strict spatial constraints on the dot-product attention calculation. This boundary restriction prevents the attention weights from spreading into background noise or adjacent features. In contrast, a sparse single-point prompt provides no spatial boundaries, leaving the attention query unconstrained. Consequently, the segmentation performance depends heavily on the internal representation capacity of the model backbone. This structural limitation causes a distinct drop in IoU scores when using SAM 1 models, where even the highly parameterized ViT-H struggles to constrain the attention field from a single click. In contrast, the newer SAM 2 architectures maintain consistently high IoU scores across all backbone scales, demonstrating that the Hiera engine handles sparse point prompts far more effectively than ViT.

**Figure 4:**
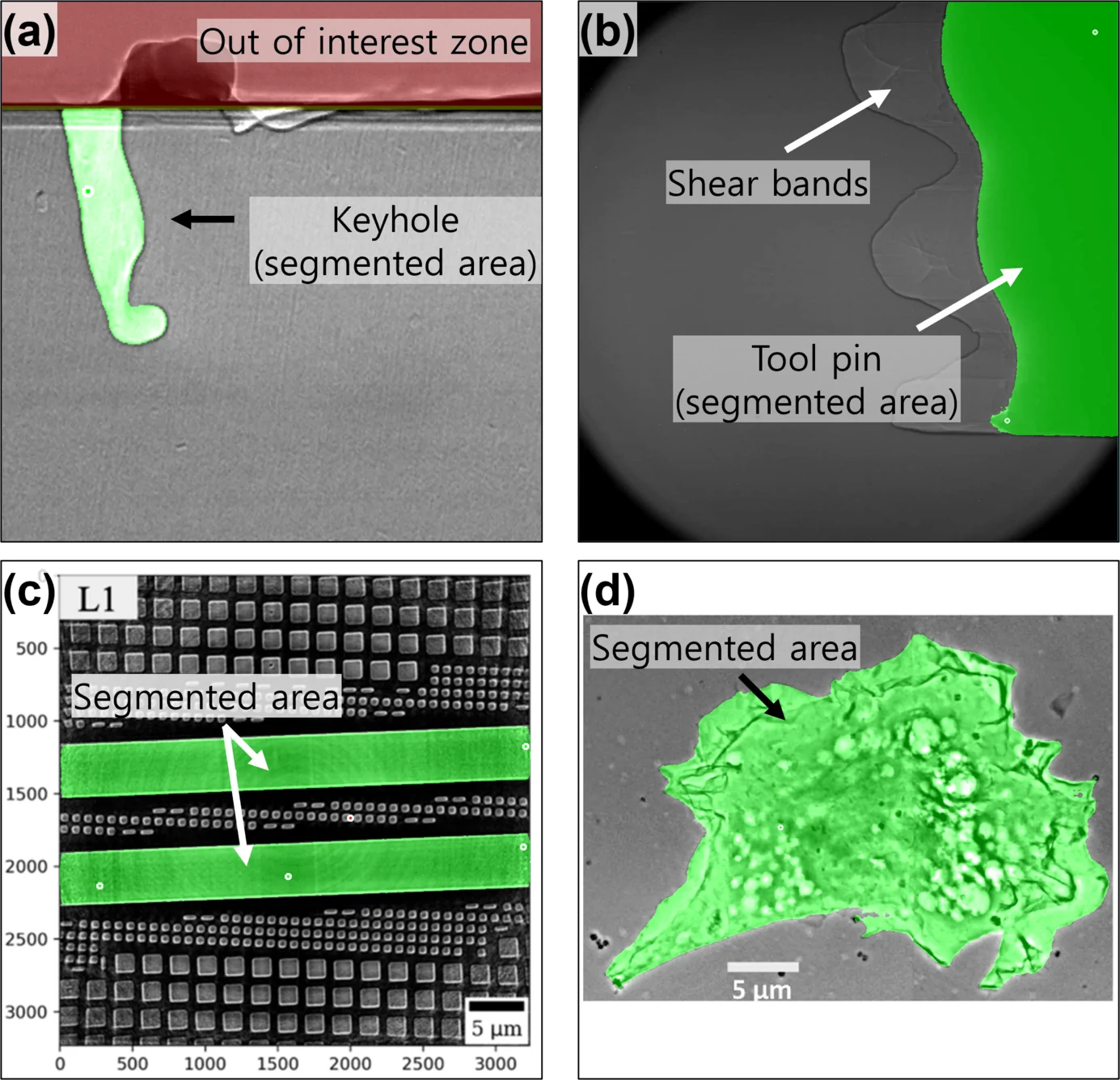
Demonstration of *SMAXI*’s interactive segmentation module applied across diverse imaging modalities and geometric complexities. (*a*) Segmentation of the keyhole in the image of an L-PBF process, utilizing an exclusion mask (red) to eliminate the out-of-interest zone above the substrate surface. (*b*) Segmentation of the tool pin in the image of a friction stir welding process (Agiwal & Pfefferkorn, 2025 ▸). (*c*) Extraction of two parallel features from the reconstructed nano-CT image of an integrated circuit (Nikitin *et al.*, 2025 ▸). (*d*) Segmentation of a biological cell (Chu *et al.*, 2008 ▸) in reconstructed CT data, demonstrating the model’s capability to autonomously capture highly irregular and complex morphological boundaries without manual pixel tracing.

The utilization of the SAM 2 (Hiera-L) segmentation module of *SMAXI* across different X-ray imaging scenarios is summarized in Fig. 4 ▸. *SMAXI* successfully handles complex geometric boundaries, such as isolating transient keyhole morphologies in operando X-ray images of the L-PBF process [Fig. 4 ▸(*a*)]. To eliminate areas that are not of interest in the segmentation tasks, *SMAXI* allows the user to define a spatial region-of-interest occlusion boundary matrix *M*_excl_. This mathematical masking suppresses anomalies arising from out-of-interest zones above the substrate surface using an elementwise Hadamard product (⊙),
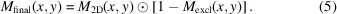
Here, *M*_2D_(*x*, *y*) ∈ {0, 1} represents the initial binary segmentation mask generated by the decoder, while *M*_excl_(*x*, *y*) ∈ {0, 1} denotes the operator-defined exclusion zone where pixels within the noisy background are assigned a value of 1. The inversion term [1 − *M*_excl_(*x*, *y*)] acts as a digital gate, evaluating to 0 inside the restricted zone and 1 within the valid experimental region. By computing the pointwise product of these matrices, any segmentation artifacts extending into the out-of-interest upper boundary are multiplied by zero and immediately suppressed, while the actual tracking geometry below remains unaltered.

*SMAXI* also adapts directly to high-visibility boundaries that do not require an exclusion mask. As demonstrated in Fig. 4 ▸(*b*), SAM cleanly isolates the rotating tool pin and tracks the adjacent material flow lines during high-speed X-ray imaging of a friction stir welding process (Agiwal & Pfefferkorn, 2025 ▸). Furthermore, the tool adapts effectively to reconstructed CT datasets. Fig. 4 ▸(*c*) shows the nano-CT image of an integrated circuit sample, in which two parallel structures are isolated in *SMAXI*. Importantly, as shown in Fig. 4 ▸(*c*), *SMAXI*’s segmentation module is not topologically restricted to generating simply connected masks. Because it leverages the zero-shot capabilities of SAM, the software can flexibly output multiple individually connected masks based on the user’s multi-point prompting strategy. This flexibility ensures that topological feature quantities, such as center-of-mass or branching network parameters, are accurately preserved for complex disjointed structures. Fig. 4 ▸(*d*) demonstrates the software’s capability of segmenting structure features with irregular shapes and rough boundaries, such as biological samples.

For volumetric 3D X-ray CT reconstruction of segmented objects, manual slice-by-slice prompting can represent an analytical bottleneck. *SMAXI* bypasses this limitation by expanding spatial features into a continuous volumetric coordinate space along the normal axis to the segmented plane (*Z* axis) via the recursive memory-conditioned block architecture of SAM. When a user introduces an initial prompt on a reference slice, a dynamic tracking memory bank is initialized to propagate this tracking state. To condition the target slice features on the geometries extracted from prior steps, a spatiotemporal memory attention block executes a cross-attention operation according to
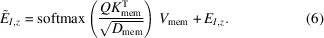
Here, *E*_*I*, *z*_ represents the raw spatial embedding of the target slice at index *z*, which acts as a residual connection to preserve the slice’s native structural details. The attention operator projects queries (*Q*) from this target slice against keys (*K*_mem_) and values (*V*_mem_) stored in the historical memory bank. The scaling factor 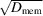 normalizes the dot-product magnitudes relative to the feature channel dimension. By applying a softmax function to the scaled matrix product 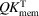, the network calculates a spatial correspondence map that highlights alignment between the new slice and previously tracked shapes. Multiplying this map by *V*_mem_ projects the historical context directly onto the current frame, yielding a memory-conditioned feature representation 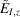 that allows the decoder to isolate boundaries without manual intervention. To recursively sustain this tracking loop along the *Z* axis, the finalized mask matrix *M*_*z*_ and its associated spatial feature maps are passed through a memory encoder (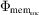). As shown in equation (7), this output is appended via a union operator ( ∪ ) to recursively update the dynamic tracking state *B*_*z*+1_ for the subsequent volume element,
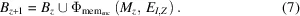
To maintain absolute structural alignment across severe physical transitions where memory attention alone might degrade, *SMAXI*’s coordinate engine extracts a precise geometric bounding box prior directly from the newly resolved mask matrix *M*_*z*_. By isolating the spatial coordinate extrema where the binary mask evaluates to 1, the system defines the tracking boundaries for the next layer via
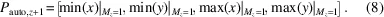
Here, *P*_auto, *z*+1_ represents the automatically generated bounding box coordinate vector designated for the upcoming slice at index *z* + 1. Mathematically, this expression scans the active pixel coordinates within the current binary matrix *M*_*z*_ where the value equals 1. It extracts the absolute minimum and maximum horizontal (*x*) and vertical (*y*) pixel indices, which correspond to the leftmost, topmost, rightmost and bottommost edges of the segmented feature. By compiling these four spatial extrema into a single vector, the system automatically constructs a tight, custom-fit bounding box around the geometry. This derived bounding box coordinate vector is automatically injected as a hard spatial prompt prior during the next evaluation step. This dual conditioning mechanism stabilizes the *Z*-axis tracking loop, with performance benchmarked across biological and material datasets.

**Figure 5:**
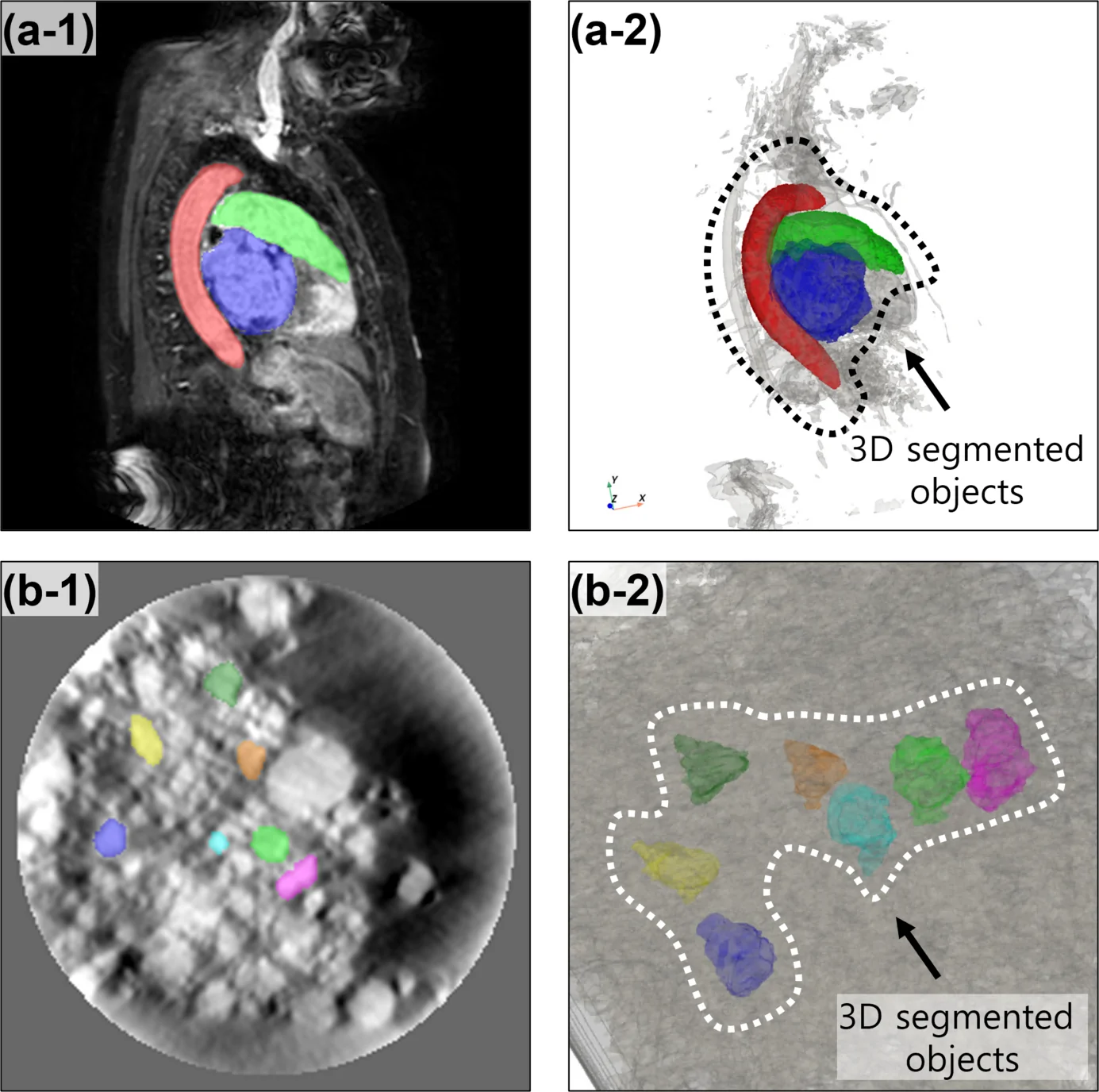
Demonstration of automated 3D volumetric propagation utilizing *SMAXI*’s memory-conditioned foundation model architecture. (*a*-1, *a*-2) A 2D cross-sectional slice and the corresponding 3D reconstructed mesh of a human heart and aorta, extracted from an *ex situ* MRI dataset via slice-to-slice propagation (Jalal, 2025 ▸). (*b*-1, *b*-2) High-fidelity segmentation and 3D rendering of discrete internal micro-features (*e.g.* porosity or secondary phases) within a micro-CT volume (De Carlo *et al.*, 2018 ▸). The algorithm successfully maintains distinct spatial identities across the *Z* axis without manual frame-by-frame intervention.

The performance of this volumetric propagation formulation is illustrated in Fig. 5 ▸. *SMAXI* demonstrates high fidelity when tracking macroscopic biological structures, converting serial *ex situ* medical slices [Fig. 5 ▸(*a*-1)] into smooth, interconnected 3D volumetric multi-organ meshes [Fig. 5 ▸(*a*-2)]. Concurrently, the algorithm tracks micro-scale engineering features, isolating discrete internal porosity networks within high-density micro-CT material scans [Fig. 5 ▸(*b*-1)]. By projecting the spatial boundaries recursively through the slice volume, *SMAXI* renders complete 3D maps of different objects [Fig. 5 ▸(*b*-2)].

#### 2.1.3. Temporal object tracking-by-detection framework (YOLO)

To track and quantify fast-moving features in *in situ* full-field X-ray imaging experiments, *SMAXI* implements a real-time tracking-by-detection module powered by a customized YOLO architecture. This module decouples the workflow into parallel spatial instance segmentation and frame-to-frame temporal association. During training, the network learns directly from high-fidelity geometric masks generated by the SAM-powered segmentation module. During inference on raw, high-speed X-ray images, the trained model bypasses slow pixel-by-pixel mask regressions by splitting the computational load into two concurrent branches. For example, to isolate a highly dynamic feature like a keyhole in L-PBF process, the first branch generates a tensor of *k* global prototype masks (*P*). *P* captures foundational shapes and structural variations across the entire frame. Concurrently, a regression branch detects the individual keyhole bounding box and predicts a corresponding vector of scalar mask coefficients *c*_*i*_. The high-fidelity tracking mask *M*_*i*_(*x*, *y*) for the keyhole is then assembled by calculating a linear combination of these global prototypes weighted by their respective coefficients, bounded by a sigmoid activation function σ as shown in
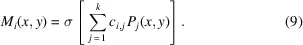
Once *M*_*i*_(*x*, *y*) are extracted, the pipeline resolves temporal tracking by associating new candidate detections (*D*_*t*_) with active historical trajectories (*T*_*t*−1_) across successive video frames. To prevent the software from mixing up tracking IDs upon different continuous frames, such as mistaking a spatter particle for the keyhole, the system matches objects between consecutive frames using a global pairing method. The spatial overlap between a new candidate detection (d) and an existing track (tr) is quantified using the bounding box IoU metric,
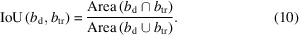
Here, *b*_d_ and *b*_tr_ represent the bounding box coordinate vectors for the candidate detection and the historical track, respectively. Using this spatial affinity, the tracking module constructs a cost matrix *C* based on the inverse tracking overlap, ensuring that highly overlapping geometries across frames yield the lowest assignment penalties,
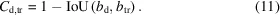
Then, the system finds the best overall pairing by minimizing the total tracking cost across all detected objects according to
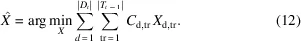
Here, *X*_d,tr_ is a simple binary switch that equals 1 if new detection d is paired with old track tr, and 0 if it is not. By multiplying these switches by the penalty scores (*C*_d, tr_) and adding them all up, the equation mathematically checks every possible pairing combination. The optimization function (argmin) then selects the single global pairing configuration (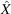), that yields the lowest possible total penalty score for the entire frame. Therefore, a newly formed keyhole is immediately assigned a new tracking ID. If the keyhole collapses or the laser shuts off, the track is automatically cleared from memory after a set number of frames. This loop ensures the software tracks only the continuous, real-time evolution of the vapor depression.

**Figure 6:**
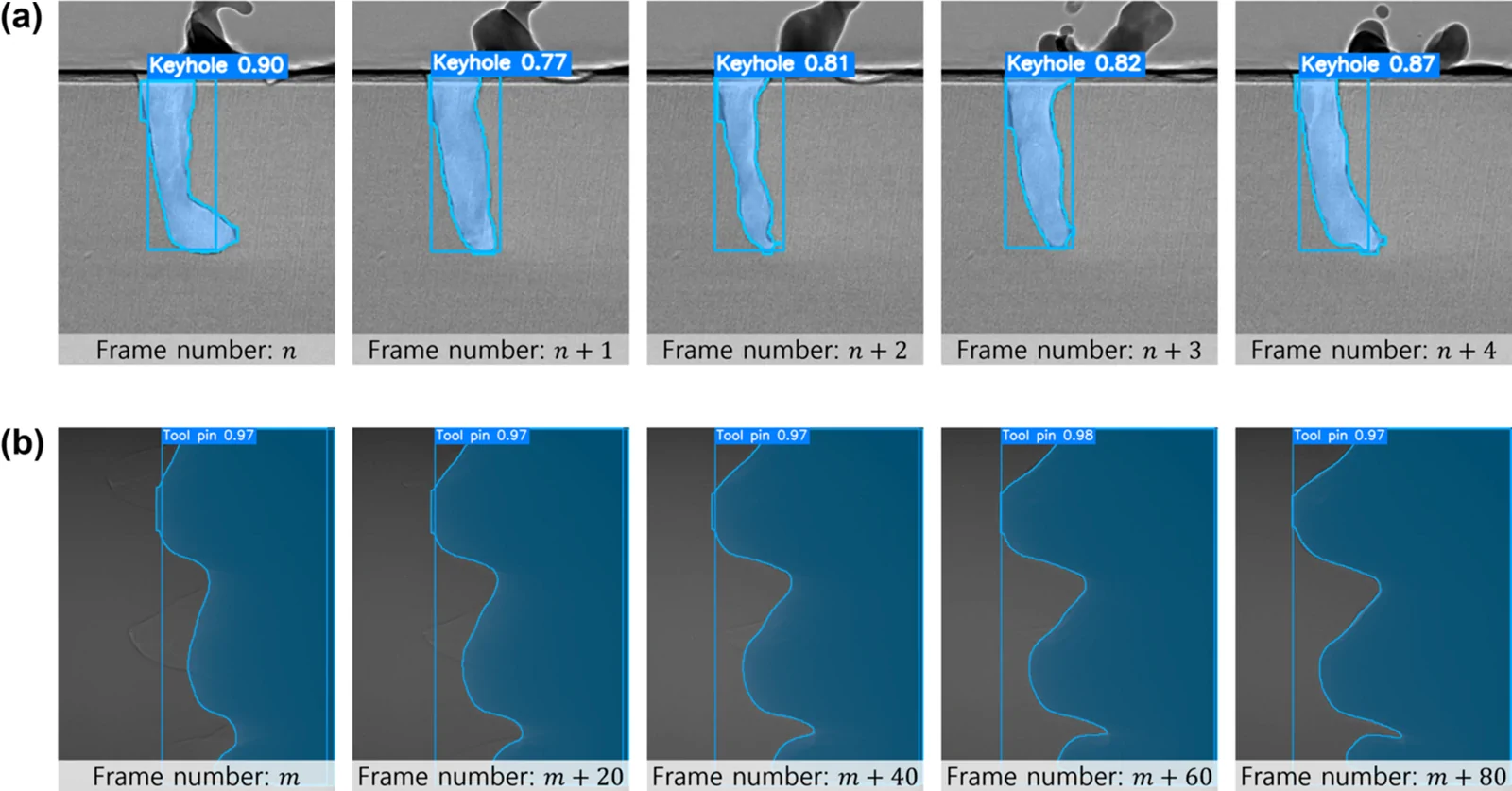
Real-time temporal tracking and instant segmentation of dynamic structure features in high-speed X-ray images using the customized YOLO module. (*a*) Tracking of a transient keyhole in the L-PBF process. (*b*) Tracking of the tool pin rotation during the friction stir welding process. Each panel displays the predicted object bounding box, the generated high-fidelity mask (blue), and the corresponding model confidence score. *n* and *m* each represents frame number of (*a*) and (*b*). Full video files are available in the supporting information.

The experimental results of the YOLO-powered real-time object tracking module are illustrated in Fig. 6 ▸. To evaluate generalization performance, each model was validated using independent X-ray image sequences that were entirely excluded from the training dataset. As shown in Fig. 6 ▸(*a*), the network successfully isolates the highly dynamic keyhole vapor depression across five consecutive X-ray image frames, maintaining a confidence score range of 0.77 to 0.90 (high-speed camera’s frame rate: 50 kHz). The coordinates of the resolved blue boundary are automatically extracted and exported into a comma-separated values (CSV) file format to calculate physical dimensions such as keyhole width, depth, aspect ratio, and total area. For the friction stir welding process shown in Fig. 6 ▸(*b*), the module tracks the rotating tool pin interface within the metal workpiece, yielding a confidence score range of 0.97–0.98 across X-ray images evaluated at 20-frame intervals (high speed camera’s frame rate: 20 kHz (Agiwal & Pfefferkorn, 2025 ▸). This higher confidence metric relative to the keyhole detection stems from the rigid structural nature of the tool pin, which exhibits minimal shape fluctuations across successive frames. In contrast, the keyhole in L-PBF process undergoes continuous geometric changes driven by local dynamic balance of the recoil pressure and Marangoni force, which increases the prediction uncertainty. The complete tracking sequences demonstrating these dynamic behaviors are provided in supplementary movies 1 and 2, respectively.

**Figure 7:**
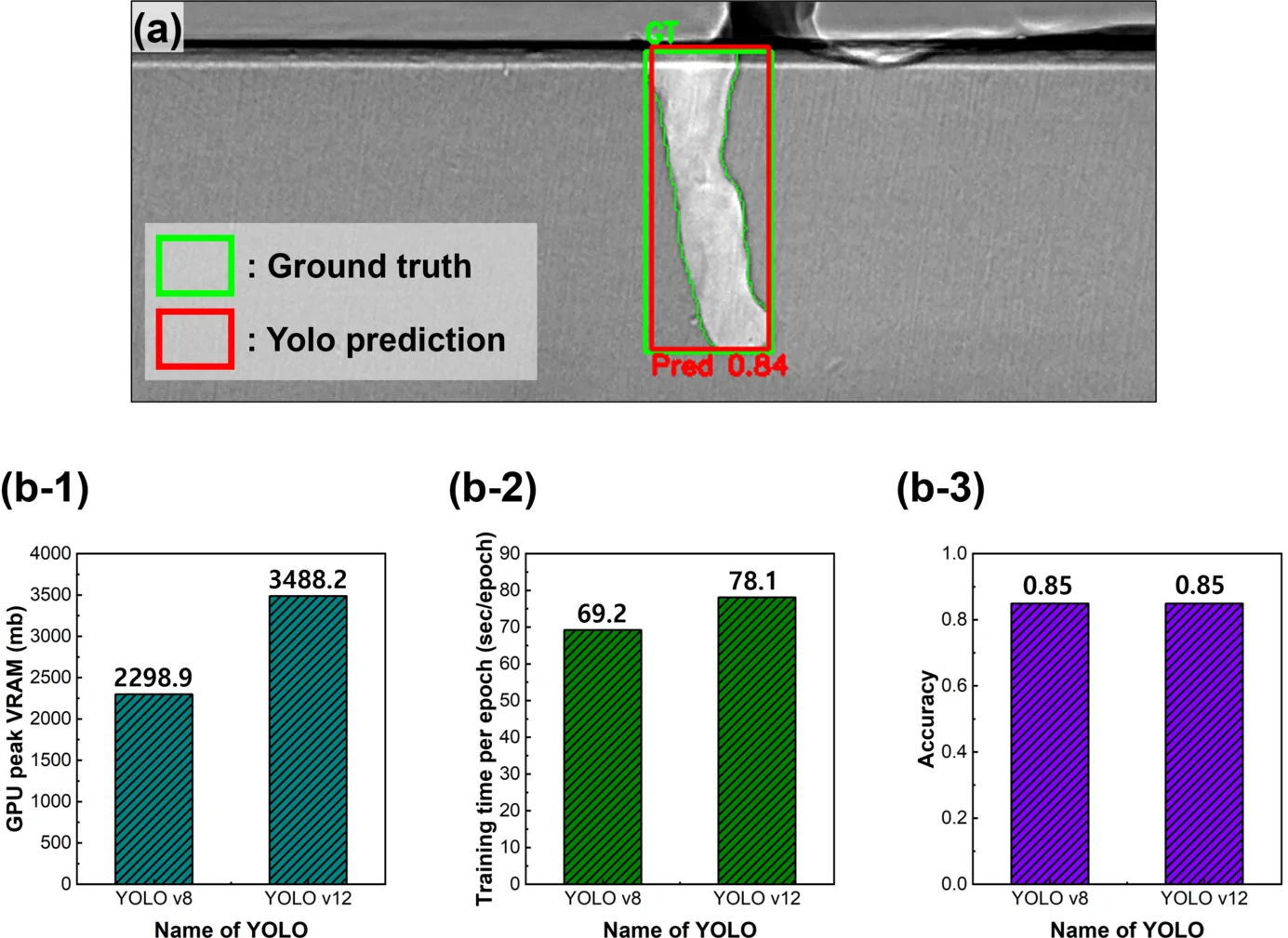
Quantitative benchmarking and performance evaluation of YOLO architectures for feature tracking in high-speed X-ray images. (*a*) Visual comparison illustrating the high spatial overlap between the manually annotated ground truth (green) and the YOLO prediction (red). (*b*-1 to *b*-3) Comparative analysis of YOLOv8 against YOLOv12. Results demonstrate that YOLOv8 requires lower peak GPU VRAM, compared to YOLOv12 (*b*-1) and demonstrates faster training times per epoch (*b*-2), and achieving equivalent segmentation accuracy (*b*-3), thereby validating YOLOv8’s integration into *SMAXI*’s object tracking pipeline.

To determine the most efficient architecture for rapid object tracking of *in situ*X-ray images, a quantitative benchmark test was conducted between two model generations: YOLOv8 and the more recent YOLOv12. As shown in Fig. 7 ▸(*a*), both architectures achieve high spatial convergence with the manually annotated ground-truth boundaries. However, newer model iterations do not automatically provide higher tracking accuracy for this application, often introducing unnecessary computational overhead that can hinder high-throughput X-ray video analysis. The resource trade-offs separating the two architectures are detailed sequentially in Fig. 7 ▸(*b*-1) through 7(*b*-3). First, looking at hardware overhead, YOLOv8 demonstrates clear advantages in memory efficiency by reducing peak GPU VRAM allocation by roughly 34.1%, requiring 2298.9 MB compared with the 3488.2 MB demanded by YOLOv12 [Fig. 7 ▸(*b*-1)]. Second, this resource efficiency translates directly to training speed, as YOLOv8 minimizes the computational workload per training epoch to 69.2 s, whereas YOLOv12 requires 78.1 s [Fig. 7 ▸(*b*-2)]. Finally, despite these differences in hardware demand and processing latency, both model variants converge to an identical structural tracking accuracy score of 0.85 [Fig. 7 ▸(*b*-3)]. These benchmarking metrics validate the selection of YOLOv8 as the optimal engine for object tracking, ensuring high-speed processing capability without sacrificing accuracy.

**Figure 8:**
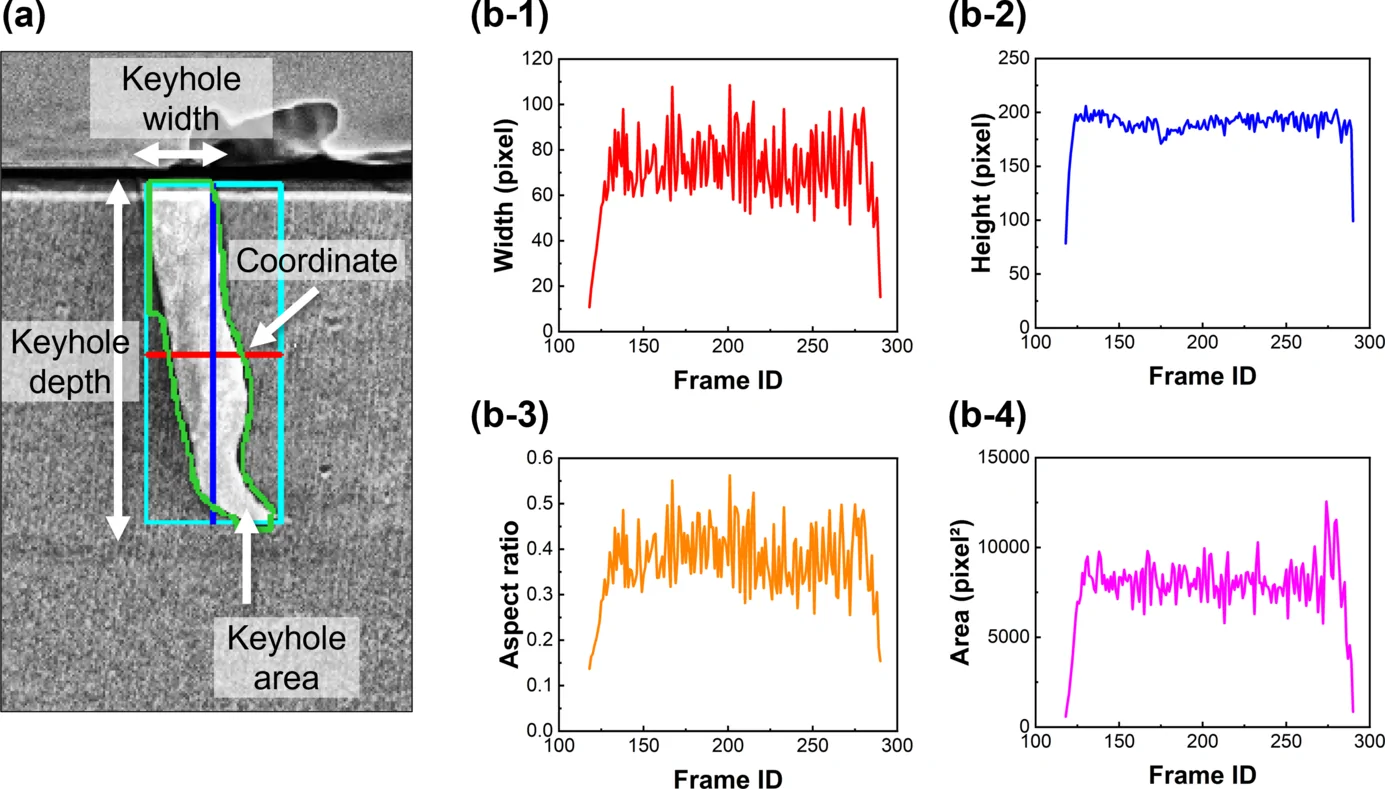
Continuous spatiotemporal analysis of keyhole morphological evolution extracted via the tracking-by-detection framework. (*a*) A tracked keyhole feature with localized dimensional axes superimposed to illustrate geometric property extraction. (*b*-1 to *b*-4) Time-resolved quantitative profiles of the extracted geometric parameters across nearly 200 consecutive frames, detailing the high-frequency fluctuations in keyhole width, height, aspect ratio, and total area. This continuous, structured data output forms the basis for subsequent LLM-driven statistical synthesis.

The final spatiotemporal dataset exported by the tracking-by-detection engine is visualized in Fig. 8 ▸. By executing the bipartite matching optimization over approximately 200 consecutive radiographs, *SMAXI* systematically maps the continuous, time-resolved morphological fluctuations of the keyhole, as demonstrated by plotting representative geometrical features. The extracted quantitative profiles are detailed sequentially in Fig. 8 ▸(*b*-1) through 8(*b*-4). First, the high-frequency oscillation profile of the keyhole width is captured over the frame sequence [Fig. 8 ▸(*b*-1)]. Second, the corresponding fluctuations in vertical height are plotted [Fig. 8 ▸(*b*-2)], which directly dictate the penetration depth of the melting zone. Third, these values are combined to trace the dynamic aspect ratio [Fig. 8 ▸(*b*-3)], a critical metric for monitoring vapor depression stability. Finally, the total projected area of the cavity is quantified to reveal the underlying energy balance driving the localized processing zone [Fig. 8 ▸(*b*-4)]. This structured tabular dataset completely replaces manual frame-by-frame digitization and serves as the direct numerical input for downstream analysis.

#### 2.1.4. LLM-driven interactive feature analysis

While the tracking module easily compresses high-throughput X-ray videos into structured numeric arrays, manually sorting through thousands of data points remains tedious. To make this tabular data immediately actionable, *SMAXI* integrates a local LLM inference engine based on the LLaMA-3 architecture. Running locally via the Ollama framework, the engine requires no external cloud Application Programming Interface (API), keeping all experimental data private and on the local workstation.

In *SMAXI*, instead of relying on computationally expensive fine-tuning of an LLM model, *SMAXI* uses context-injected prompt engineering to help the model interpret the numeric datasets. Users provide a raw time-resolved geometric feature matrix generated by the YOLO module as *D* ∈ *R*^*N*×*M*^, where *N* is the number of video frames and *M* represents the extracted attributes like keyhole width, depth, area, and aspect ratio. Before opening the user interface, a serialization operator *T* calculates a column-wise statistical summary matrix *S* containing the mean, standard deviation, and min/max boundaries for every tracking metric. The operator formats both the raw frame data and these statistical limits into a text-based Markdown string, *S*_data_ = *T*(*D*, *S*). When a user inputs a natural language question (*Q*_user_), *SMAXI* combines it with a system prompt (*P*_sys_) that sets the physics domain rules and units. The final joint token sequence (*T*) fed into the transformer network is concatenated,
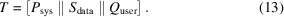
Here, the ∥ operator simply means the text blocks are glued together back-to-back into a single prompt. This ensures the model receives the physical rules, the raw data values, and the user’s question all at once. The model then processes this sequence through its internal attention layers to generate the final response sequence (*R*_LLM_) shown in 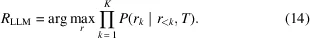
Conceptually, equation (14) represents how the autoregressive model predicts the next most logical word (*r*_*i*_) one by one. By conditioning each new word on the entire input sequence (*T*) and all previously generated words, the equation mathematically forces the LLM to ground its qualitative analysis strictly within the boundaries of the empirical feature matrix *D*. This prevents the model from hallucinating false physical trends.

**Figure 9:**
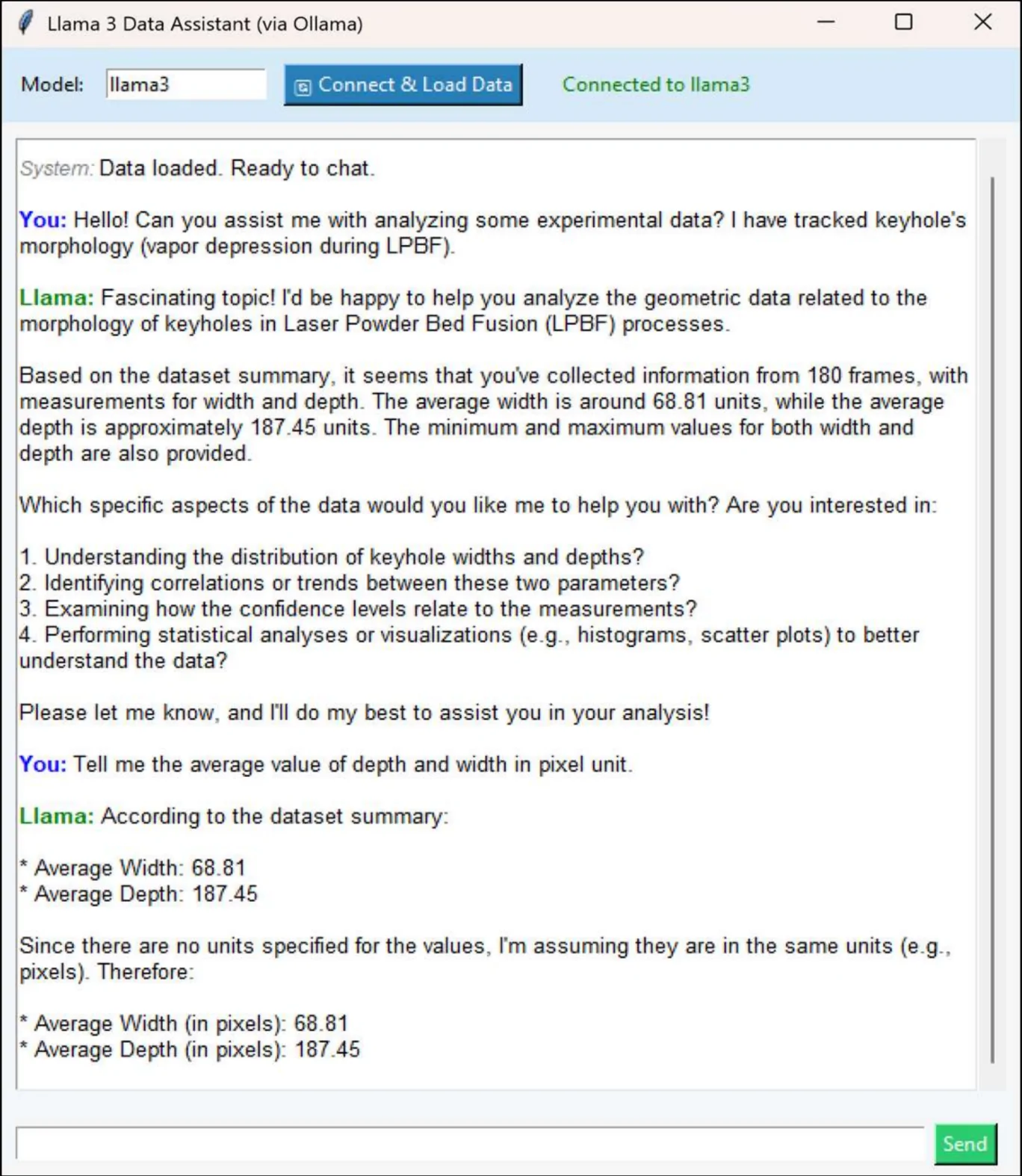
GUI of *SMAXI*’s LLM-driven interactive data assistant module. The local interface integrates a LLaMA-3 transformer model via the Ollama framework to parse the structured spatiotemporal datasets generated by the tracking engine. The interaction profile demonstrates the model’s capacity to ingest a serialized 180-frame L-PBF keyhole morphology dataset, interpret the system metadata context, and execute immediate, human-readable natural language statistical summaries regarding transient keyhole width and depth metrics.

The practical software implementation of this conversational data synthesis layer is visualized in Fig. 9 ▸. Upon loading a generated 180-frame tracking dataset (*N* = 180) derived from an *in situ* L-PBF experiment, the graphical interface of the framework successfully establishes a secure connection to the locally hosted LLaMA-3 instance. As demonstrated by the chat history, the localized metadata serialization layer successfully injects the context of the keyhole matrix. The model autonomously reads the correct context parameters, identifying the total frame volume and accurately isolating the statistical mean values for the keyhole dimensions (μ_width_ = 68.81 and μ_depth_ = 187.45 pixels). When prompted with follow-up semantic requests regarding pixel dimensions, the model displays robust contextual continuity. This conversational architecture also allows advanced statistical parsing, transforming large tabular X-ray datasets into immediate quantitative numbers.

### 2.2. Auxiliary functions of *SMAXI*

While the core machine learning pipeline handles the primary normalization, segmentation, tracking, and LLM-based quantitative analysis tasks, *SMAXI* incorporates auxiliary modules to ensure *SMAXI*’s data fidelity and support user-driven customization of the software.

#### 2.2.1. Computer vision-based image height stabilization

High-speed synchrotron experiments and long-duration laboratory X-ray scans frequently introduce unwanted spatial artifacts due to sub-micron mechanical vibrations, stage instabilities, or localized thermal expansion, such as the rapid physical expansion of the substrate during L-PBF processes. These minor translational shifts distort the coordinate system across sequential images, and thereby introduce geometric errors in the background-removal-based image normalization algorithm discussed in Section 2.1.1. To counteract these spatial misalignments without altering raw pixel distributions, *SMAXI* features a rigid translational stabilization module. This computer vision-assisted module operates by calculating the optimal spatial displacement vectors through a normalized cross-correlation algorithm evaluated against a designated reference frame. Let *I*_raw_(*x*, *y*, *t*) denote the raw intensity of a pixel at spatial coordinates (*x*, *y*) within a frame captured at time step *t*, and let *I*_raw_(*x*, *y*, *t*_0_) represent a stationary baseline reference frame or an optimized region of interest selected at initial time *t*_0_. The vertical translation correction scalar, Δ*y*(*t*), required to realign the frame sequence, is determined by maximizing the cross-correlation surface over a discrete pixel search domain,
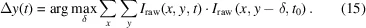
Here, the search parameter (δ) represents the vertical pixel shift window applied to the reference frame. By finding the value of δ that yields the highest mathematical overlap between the current frame and the baseline, the equation isolates the exact magnitude of the vertical shift. Once this displacement offset is quantified, the spatial coordinate space of the current image matrix is uniformly realigned via a rigid translation. The finalized, spatially stabilized output matrix *I*_stab_ is mathematically formalized as[Disp-formula fd16]
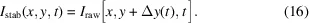
By isolating and eliminating physical mechanical jittering movement from the frame sequence, the accuracy of the subsequent background removal image normalization process is improved.

#### 2.2.2. *SMAXI* customization: module integration

**Figure 10:**
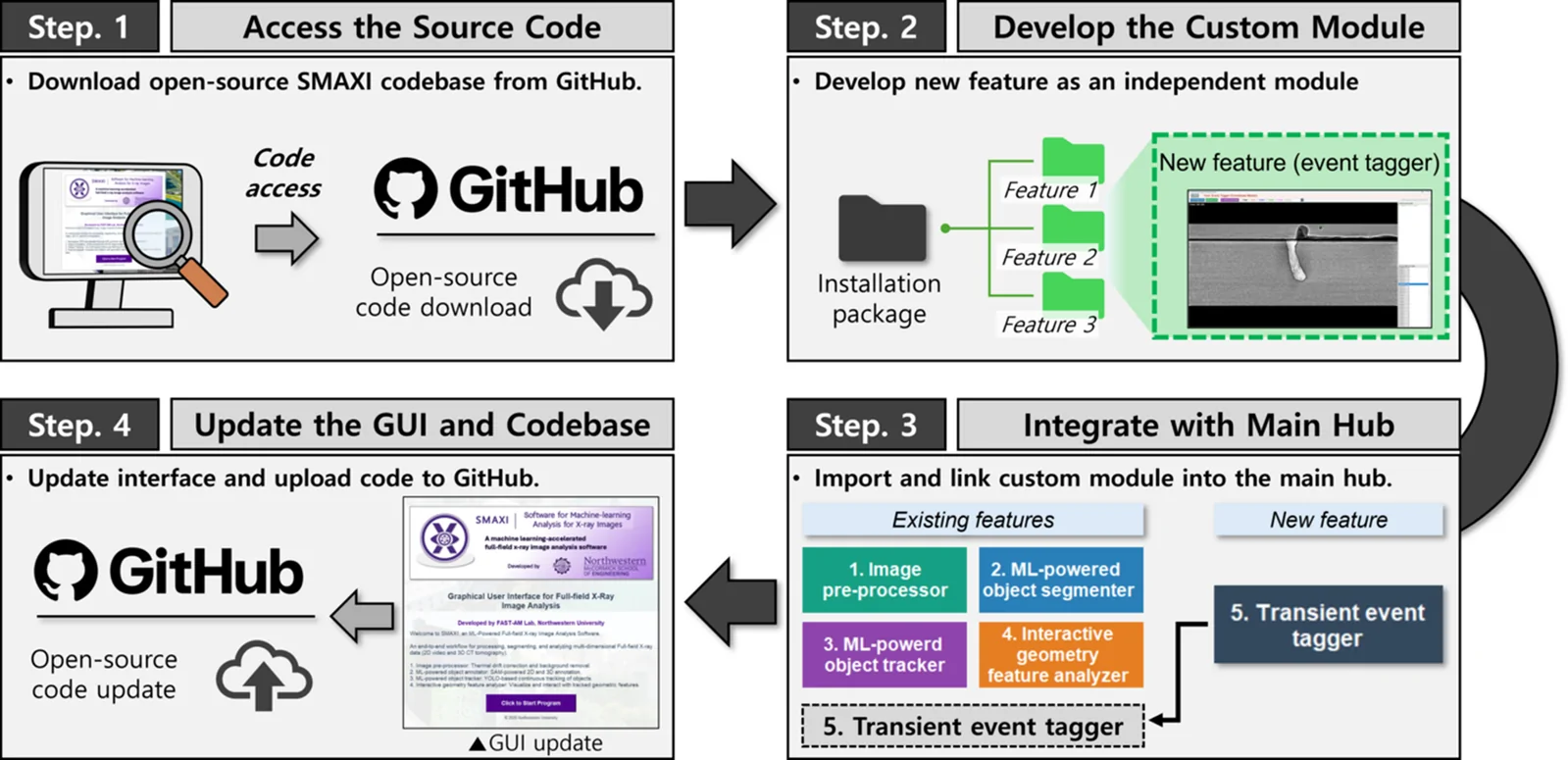
Overview of the modular expansion and open-source integration framework within *SMAXI*. The four-stage workflow illustrates the systematic development of the lifecycle for expanding the platform’s native features, using the integration of an auxiliary transient event tagger module as a case study. The protocol encompasses initial GitHub codebase access (Step 1), independent module design (Step 2), registration and linking within the main hub (Step 3), and final GUI view updates for deployment back to the open-source community via GitHub (Step 4).

*SMAXI* is fundamentally engineered around an object-oriented, highly decoupled software architecture designed to ensure long-term extensibility for the advanced X-ray imaging community. Recognizing that distinct experimental configurations necessitate highly tailored computational solutions, the platform establishes a structured, standardized blueprint for integrating custom user-developed features without risking codebase regression or architecture fracturing. The modular expansion lifecycle implemented within *SMAXI* comprises a systematic four-stage development pipeline, as schematically mapped in Fig. 10 ▸.

*Step 1 (accessing the open-source codebase)*. Users download the open-source *SMAXI* code directly from GitHub (Kim, 2026 ▸). This gives them full access to all the software files, user interface code, and data analysis tools.

*Step 2 (independent module development)*. Using *SMAXI*’s standardized templates, users can build and add their own analysis tools as independent Python code. As an example, a custom ‘Transient Event Tagger’ module, allowing users to click directly on the X-ray video frames to visually mark and map temporary processing anomalies, can be created.

*Step 3 (integration with the central core hub)*. Once validated independently, the new sub-package is imported into the central hub script. The developer registers the custom module alongside the four baseline execution frameworks, the image pre-processor, the ML-powered object segmenter, the temporal tracker, and the natural language feature analyzer.

*Step 4 (GUI view restructuring and deployment)*. Finally, the GUI view layout is updated by appending the appropriate execution button hooks and interactive window elements onto the master window class. The updated, compiled tool is then pushed back to the open-source GitHub ecosystem, expanding the platform’s utility and fostering community-driven development.

## 3. Conclusion

In summary, the broader application of full-field X-ray imaging techniques and the rapidly increasing rate of data generation demand efficient and effective software for image processing and analysis. To address this challenge, *SMAXI* was developed with functions designed to handle large-volume multiscale X-ray radiography and CT data. By integrating advanced ML frameworks and localized LLM models into a unified, open-source environment, *SMAXI* replaces labor-intensive workflows with an automated, high-throughput pipeline. The operational efficacy of *SMAXI* is driven by four core integrated technological features, which were successfully demonstrated using representative X-ray image datasets.

*Feature 1*. A pre-processing engine that executes percentile-based continuous gray-scale normalization and auxiliary translational drift stabilization was developed to deliver image contrast enhanced X-ray images for successful segmentation and tracking tasks.

*Feature 2*. An interactive 2D boundary isolation and automated 3D volumetric propagation framework leveraging foundation vision models (SAM 1 and 2) were developed to eliminate manual pixel segmentation tasks across large image stacks.

*Feature 3*. A real-time tracking-by-detection module powered by the YOLO framework was integrated into *SMAXI*. This allows the software to lock onto specific shapes and maintain their tracking identities across continuous video frames.

*Feature 4*. A data-private, locally deployed LLM interface that ingests serialized morphological datasets was integrated into *SMAXI*, thus allowing users to query complex spatiotemporal trends through intuitive conversational queries.

*SMAXI* stands out as a powerful open-source image analysis software for the global X-ray community, delivering rapid analysis results to accelerate the pace of scientific discovery incorporating full-field X-ray imaging. While *SMAXI* currently provides a highly robust, zero-shot framework for segmenting and tracking distinct morphological features, it is important to note the limitations of its current foundational backbones. At the time of writing, models such as SAM and YOLO can struggle with tracking accuracy during instances of object occlusion or in datasets with extremely low signal-to-noise ratios. However, *SMAXI* is fundamentally designed as an extensible platform. As foundational models rapidly evolve, its modular architecture will allow for the seamless integration of updated backbones. Furthermore, we plan to expand the integrated LLM feature into an autonomous AI agent capable of executing the entire pipeline through natural language. This will transform *SMAXI* into a fully query-based software where users can conduct pre-processing, tune segmentation parameters, and track workflows via conversational prompts. Ultimately, we intend for *SMAXI* to grow through continuous community-driven contributions and collaborations that welcome the integration of all domain-specific physics plugins, customized data filtering algorithms, and advanced 3D reconstruction modules, such as *TomocuPy* (Nikitin, 2023 ▸). In doing so, we hope *SMAXI* will serve not only as a practical research tool, but also as a shared foundation for future innovations in X-ray image analysis, data science and AI-assisted scientific discovery.

## Supplementary Material

Supporting Video S1 relating to Fig. 6. DOI: 10.1107/S1600577526007745/tv5094sup1.mp4

Supporting Video S2 relating to Fig. 6. DOI: 10.1107/S1600577526007745/tv5094sup2.mp4

## Figures and Tables

**Table 1:** Different types of SAM utilized for object segmentation (Kirillov *et al.*, 2023 ▸)

Type	Name of SAM	Description
SAM 1	ViT-H (ViT-Huge)	Most accurate SAM 1 model. Ideal for highly precise segmentation. Requires high computational power and runs slower.
	ViT-L (ViT-Large)	Balances processing speed and accuracy. Ideal for standard 2D X-ray images and moderate CT data.
	ViT-B (ViT-Base)	Fastest SAM 1 model. Requires low computational resources. May have slightly lower accuracy on complex or low-contrast features.

SAM 2	Hiera-L (Hiera-Large)	Most advanced SAM 2 model. Best for highly challenging segmentation tasks. Ideal for massive CT data requiring maximum accuracy.
	Hiera-S (Hiera-Small)	Lightweight model. More accurate than the Tiny version. Maintains high speed for dynamic video data.
	Hiera-T (Hiera-Tiny)	Most compact SAM 2 model. Highly optimized for speed. Best for tracking fast objects in massive *in situ* X-ray videos.

## Data Availability

The *SMAXI* source code is openly available via GitHub at https://github.com/dukyong414/SMAXI. Sample multidimensional X-ray image datasets reported in this article are included directly within the repository’s data directory or hosted publicly on TomoBank and Mendeley Data.

## Appendix A SAM for image segmentation tasks

Appendix *A* explains the architecture and mechanism of SAM in segmentation tasks. The SAM-powered interactive segmentation decomposes the processing workflow into three consecutive operators, an image encoder (Φ_image_), a prompt encoder (Φ_prompt_), and a two-way cross-attention mask decoder (Φ_decoder_) (Kirillov *et al.*, 2023 ▸). First, the Φ_image_ utilizes a ViT backbone to capture the global structural context of the pre-processed X-ray slice. The input image matrix is partitioned into a sequence of *N* non-overlapping spatial patches of size *P* × *P*. These patches are flattened, linearly projected into a latent space of dimension *D*, and summed with learnable 2D positional embeddings to preserve the original spatial topology of the X-ray image. This token sequence is then processed through consecutive transformer layers to yield a dense spatial feature embedding *E*_*I*_ representing the entire field of view. Concurrently, the Φ_prompt_ translates user-guided coordinate inputs from the graphical interface into sparse tokens. Foreground/background click coordinates are mapped into positional tokens using Fourier features γ(*x*, *y*) and summed with learned state vectors. For bounding boxes, the boundary conditions are encoded by concatenating the positional mappings of the top-left and bottom-right corners, producing a sparse token sequence *E*_*P*_. To construct the finalized feature mask, the lightweight Φ_decoder_ executes a two-way cross-attention mechanism that iteratively mixes the sparse prompt tokens and dense image embeddings. In this layer, the prompt embeddings are mapped as queries (*Q*), while the spatial image features act as keys (*K*) and values (*V*). The scaled dot-product attention function evaluates the localized spatial correspondence between the user’s intent and the global feature field,

By applying a pointwise softmax activation function to the scaled dot product of the *Q* and *K*, the network assigns higher contextual weights to image regions that align with the spatial prompts, ensuring sharp topological constraints for the final mask computation. Following this attention-driven weighting step, the refined prompt tokens project a dynamic classifier weight vector (**w**). This vector undergoes a spatially dense dot product with the upscaled image feature maps to generate raw mask logits (*L*). The final binary 2D segmentation mask (*M*_2D_) is then isolated by passing these logits through a point-wise sigmoid activation function (σ) subject to a predefined confidence threshold (τ),

This mathematical formulation allows *SMAXI* to reliably capture irregular or low-contrast boundaries. By defining features through geometric constraints rather than manual drawing-based segmentation, the platform standardizes the segmentation process and removes subjective operator bias.
